# Morphological and biomechanical characterization of long bones and peri-implant bone repair in type 2 diabetic rats treated with resveratrol

**DOI:** 10.1038/s41598-024-53260-4

**Published:** 2024-02-04

**Authors:** Carolina Sayuri Wajima, Letícia Pitol-Palin, Fábio Roberto de Souza Batista, Paulo Henrique dos Santos, Doris Hissako Matsushita, Roberta Okamoto

**Affiliations:** 1 Department of Basic Science, Araçatuba School of Dentistry, São Paulo State University (UNESP), Araçatuba, São Paulo Brazil; 2 Department of Diagnosis and Surgery, Araçatuba School of Dentistry, São Paulo State University (UNESP), Araçatuba, São Paulo Brazil; 3 Faculty of Dentistry - Restorative Dentistry Area, University of Toronto, Toronto, Canada

**Keywords:** Diseases, Endocrinology, Risk factors, Signs and symptoms

## Abstract

Type 2 diabetes interferes with bone remodeling mechanisms, requiring studies to reverse this damage, and resveratrol is a polyphenol with rich properties. This study aimed to characterize the long bone morphology and peri-implant biomechanics of normoglycemic and type 2 diabetic animals treated with resveratrol. Thirty-two male Wistar rats were used and divided into normoglycemic and diabetic with or without treatment. They had the installation of implants in the tibia and treatment with oral resveratrol within 45 days. Resveratrol was responsible for weight homeostasis and decreased glycemic levels in rats with type 2 diabetes. The three-point bending testing, resveratrol showed positive effects on the biomechanics of long bones, corroborating a more resistant bone in comparison to untreated diabetics. Micro-ct revealed how bone metabolism is affected by systemic disease, decreasing bone quality. The counter-torque of normoglycemic animals showed superior osseointegration to diabetes, with no differences in the administration of the polyphenol, showing the sovereignty of the deleterious effects of the disease when there is a tissue lesion and an inflammatory picture installed. Overall, resveratrol acted positively in the etiopathogenesis of type 2 diabetes and revealed positive effects on the strength of long bones.

## Introduction

In an increasingly obese population, with poor eating habits, resulting in an unhealthy lifestyle, type 2 diabetes becomes a major global public health problem, with 463 million people with diabetes, where 50.1% of adults are undiagnosed and type 2 is responsible for 90–95% of patients^1^. This is characterized by insulin resistance. As there is no proper functioning, glucose levels continue to rise and release more insulin, insufficient to restore normal levels and generate hyperglycemia^2^. It acts multi-systemically and with great comorbidity, promoting cardiovascular, retinal, renal, and neurological complications^3^, and also acting on bone health, which according to the Brazilian Society of Diabetics is not infrequently neglected^4^.

Individuals with type 2 diabetes have lower bone quality and bone strength, being at a higher risk of bone fractures, where high blood glucose levels damage the mineral matrix, through altered cellular function and bone remodeling and an accumulation of advanced glycation end products (AGEs)^5^, The pathological effect of AGEs is related to their ability to modify physical and chemical properties of various structures^6,7^, causing deterioration of collagen and changes in bone microstructure^8,9^. In the literature, besides type 2 diabetes being associated with an increased risk of fractures, there is also an influence on oral health. The oral cavity is compromised through the increase of infections, bone loss, and periodontal disease, promoting a delay in remodeling and alveolar bone repair^10,11^, and especially tooth loss^12^. According to the World Health Organization, type 2 diabetes is late diagnosed due to its silent symptoms, and is often discovered in middle age, where, in dentistry, there is a higher prevalence of implant installation. Understanding the structural characteristics and reversing the pathophysiological changes of bone is of great importance to increase the success of rehabilitative treatment, since diabetes leads to a greater loss of implants when compared to normoglycemic patients^13^.

Due to this condition, studies have been searching for alternative therapies, such as herbal medicines, which lead to favorable results without side effects. Resveratrol is a polyphenol (3,5,4ʹ-trihydroxy-trans-stilbene), typically found in the skin of purple grapes, wines, and other plants^14^. It has anti-inflammatory, antioxidant, cardioprotective, and antidiabetic properties^15^. It has been a widely accepted choice for the treatment of type 2 diabetes by preventing glucose intolerance, significantly decreasing body weight, and body mass index in obese people, and increasing lean body mass^16^. Studies have demonstrated the beneficial effect of resveratrol on bone tissue, preventing bone loss, acting on structures, volume, and bone microarchitecture^15^, and also showing effects on post-surgical repair^14–17^. Thus, the aim was to characterize the long bones from the morphological and functional point of view, and the peri-implant biomechanics of normoglycemic and type 2 diabetic animals, treated or not with resveratrol.

## Methods

This study was approved by the Ethics Committee on the Use of Animals (CEUA) of São Paulo State University (UNESP), School of Dentistry, Araçatuba, Brazil (#00153-2019, approved in February 15th, 2019), following the Animal Research: Reporting of In Vivo Experiments (ARRIVE) guidelines, and following the Guide for the Care and Use of Laboratory Animals of the National Institutes of Health (Institute of Laboratory Animal Resources [U.S.])^18^.

### Animals

Thirty-two male rats (*Rattus norvegicus albinus*, Wistar), weighing an average of 395 g and aged 3 months, were divided into four groups (n = 8 per group): Normoglycemic (NG); Normoglycemic + resveratrol (NGrvt); Type 2 diabetes (T2D); and Type 2 diabetes + resveratrol (T2Drvt). The animals were kept in cages in a stable temperature environment (22 °C ± 2 °C, light control cycle 12 h light, 12 h dark) and a balanced diet (NUVILAB, 1.4% Ca and 0.8% P + water with *libitum*) based on previous results already published^10^: the averages used for the calculation were 3.06 and 4.898 and the standard deviations were 0.26 and 0.024, with a significance level of 5% and a power of 95% in a one-tailed hypothesis test. The animals were identified by numbers and randomly separated by Microsoft Office Excel software (Microsoft, Redmond, WA, USA), respecting a 1:1 allocation rate for each group. The data obtained from the proposed analyses were submitted to statistical analysis by the software GraphPad Prism 7.01 (GraphPad Software, La Jolla, USA).

### Type 2 diabetes induction

**Figure 1:**
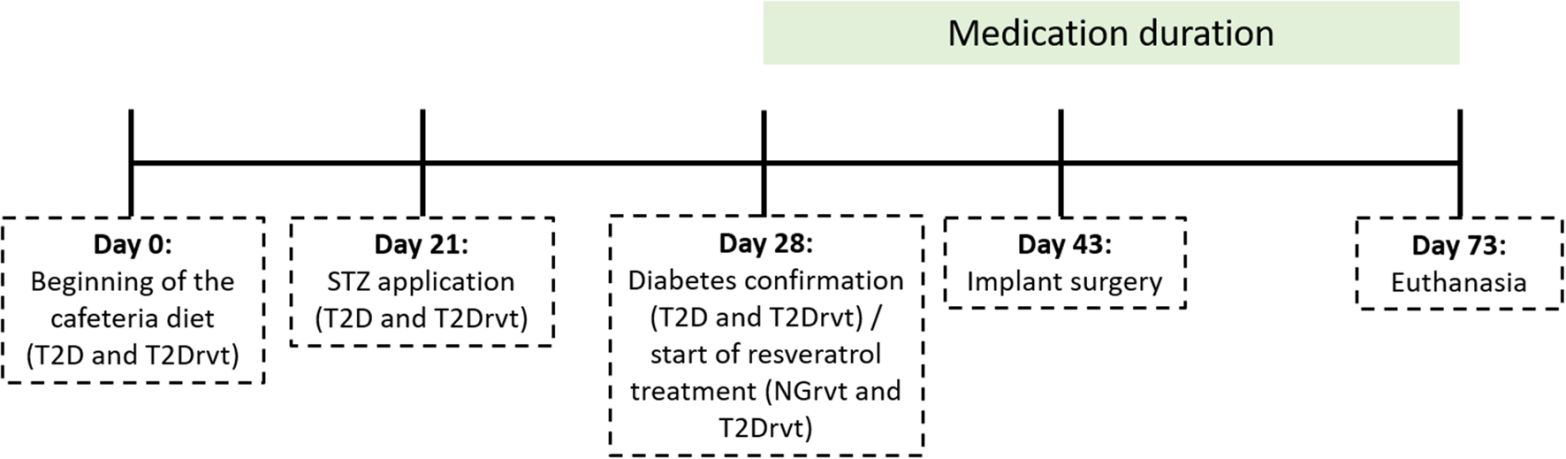
Experimental model timeline. Beginning of the diet = day 0; STZ application = day 21; the type 2 diabetes confirmation and the start of resveratrol treatment = day 28; implant surgery = day 43 and euthanasia = day 73.

The combination of the cafeteria diet associated with low-dose streptozotocin (STZ) application was used only in T2D and T2Drvt groups. The cafeteria diet followed the model of Gomez-Smith^19^ and Carillon^20^, mimicking a poor diet that promotes insulin resistance and obesity in rats, which are predisposing factors for type 2 diabetes^20^. The cafeteria diet started on day 0 and each animal received 30 g of this diet, consisting of 10 g of stuffed crackers, 10 g of wafer crackers, 10 g of corn snacks, and a bottle of sugar water with a concentration of 12% sucrose (50 mL daily) (Table 1). After 3 weeks of the cafeteria diet, drug induction of type 2 diabetes occurred through the Srinivasan model^21^, where animals of T2D and T2Drvt groups, were anesthetized by intramuscular infiltration of 5 mg/kg xylazine hydrochloride (Dopaser ®—Laboratórios Calier do Brazil Ltd.a., Osasco, Brazil) and 50 mg/kg ketamine hydrochloride (Vetaset®—Fort Dodge Animal Health Ltd.a., Campinas, Brazil), antisepsis of the scrotal region was done with 70% alcohol and a low dose of streptozotocin (STZ—Sigma©, Merck KGaA, Darmstadt, Germany and/or its affiliates) was injected into the penile vein (35 mg/kg), dissolved in a vehicle (0.1 M sodium citrate solution, pH = 4.5) and the NG and NGrvt animals received only the vehicle. The low dose STZ used in this study causes partial destruction of beta cells and accelerates the development of the type 2 diabetes condition^10,19^. In specific and strategic data, the animals were weighed on a conventional scale and their glycemic levels were measured. Periods: the beginning of the diet (day 0), STZ application (day 21), the type 2 diabetes confirmation and the start of resveratrol treatment (day 28), implant surgery (day 43), and euthanasia (day 73). To perform the glycemic evaluation, the animals were fasted for 2 h after consuming the oral solution containing glucose (2 g glucose), and, using a n°11 scalpel blade a blood sample was taken from the caudal vein through a small perforation. The blood sample should be a compatible drop for reading the glucose meter^22^. Type 2 diabetes was determined with concentrations greater than 198 mg/dL after 2 h of oral glucose administration^22,23^. This study model showed that a cafeteria diet and a low dose of STZ are effective in generating an animal model that reproduces a common type of natural and metabolic history of diabetes 2 in humans^19^ (Fig. 1).

**Table 1:** Cafeteria diet.

Foods	Amount (per rat)
Stuffed crackers	10 g daily
Wafer crackers	10 g daily
Corn chips	10 g daily
Water with sugar (12%)	50 mL daily

Cafeteria Diet. Foods that make up the cafeteria diet (T2D and T2Drvt): Stuffed crackers, wafer crackers, corn chips, and also a bottle of water with a concentration of sugar (12%).

### Resveratrol treatment

After confirmation of type 2 diabetes (day 28) in animals of T2D and T2Drvt groups, the systemic treatment with resveratrol (Aphoticário Pharmacy of Manipulation Ltda, Araçatuba, São Paulo, Brazil) was started in animals of NGrvt and T2Drvt groups until the end of the experiment, totaling 45 days of treatment. It was administered orally, by gavage technique, at a concentration of 100 mg/kg/day dissolved in Dimethyl Sulfoxide (DMSO)^23^ and the animals in NG and T2D groups, to undergo the same stress, received only the vehicle.

### Implant surgery

The surgery to install implants (day 43) was performed fifteen days after the beginning of resveratrol treatment, the animals were forced to fast for 8 h where animals of all and then sedated, as described previously. The medial portion of the left tibia was trichotomized and the region to be incised was treated with antiseptic. With a number 15 blade, an incision was made in the region of the left tibial metaphysis, and then the soft tissue was divulsion in its total thickness and detached, exposing the bone to receive the implants.

Each animal received 1 implant (Medens, São Paulo, Brazil), in the region of the left tibial metaphysis, with a total of 32 titanium implants with double acid etched, 2.0 mm in diameter, and 4.0 mm in height, sterilized by gamma rays. For this purpose, the milling will be performed with a 1.6 mm diameter spiral cutter mounted on an electric motor (BLM 600®; Driller, São Paulo, SP, Brazil), at a speed of 1000 rpm, under irrigation with isotonic 0.9% sodium chloride solution (Fisiológico®, Laboratórios Biosintética Ltda®, Ribeirão Preto, SP, Brazil), and counter-angle (Angular Part 3624N 1:4, Head 67RIC 1:4, KaVo®, Kaltenbach & Voigt GmbH & Co, Biberach, Germany), with a 16:1 reduction and a depth of 3.0 mm, with initial blocking and stability. The tissues were sutured in planes with absorbable thread with continuous stitches in the deep plane and with monofilament thread with interrupted stitches in the outermost plane. In the immediate postoperative period, each animal received a single intramuscular dose of 0.2 ml of veterinary antibiotic (Pentabiotic Small Veterinary, Fort Dodge Saúde Animal Ltda., Campinas, São Paulo, Brazil) and analgesia with Dipyrone Sodium (1 mg/kg/day, (1 mg/kg/day, Ariston Indústrias Químicas e Farmacêuticas Ltda, São Paulo, Brazil)^24^.

### Sample processing

Animals were euthanized by an anesthetic overdose of xylazine hydrochloride and ketamine hydrochloride 30 days after implant installation, with the tibia still in vivo, and counter-torque analysis was performed. The femurs were collected and fixed in formaldehyde 10% for 48 h, then washed in running water for 24 h and immersed in alcohol at 70° at room temperature, to be scanned for computed microtomography analysis. The femurs used for Three-points bending testing were removed, washed in saline to remove blood and immediately, conserved in saline at the refrigerator. Only on the day of the biomechanical tests were the specimens thawed.

### Biomechanical analysis of counter-torque

The animals were euthanized by anesthetic overdosage of xylazine hydrochloride (Dopaser®—Laboratórios Calier do Brazil Ltda., Osasco, Brazil) and ketamine hydrochloride (Vetaset®—Fort Dodge Animal Health Ltd.a., Campinas, Brazil) 30 days after the implant installation. The tibia was accessed to expose the implants and a digital torque meter (Homis, São Paulo, SP, Brazil) coupled with a 1.2 mm hexagonal digital key was used (Medens, São Paulo, SP, Brazil). A counterclockwise motion was applied until rotation of the implant within the bone tissue and complete rupture of the bone/implant interface. The maximum value for rupture was recorded on the torque meter (N.cm) and noted^25^.

### Three-points bending testing

Biomechanical tests provide some properties of bone tissue^26^, given when stimulus are applied to bone and the intensity of this stimulus necessary to break the bone as well as the behavior of the bone when is broken are evaluated. All of these parameters are measured through: Maximum force (N) that corresponds to the greatest force to completely break the femoral bone, obtained in the region of the femoral shaft. Rupture force (N) corresponds to the force required to break the bone marrow area of the femurs. The deformation at maximum force (mm) corresponds to how much the bone deforms during the application of the maximum force and, finally, the deformation at rupture (mm), which corresponds to the deformation of the bone at the moment of rupture. The test was performed on the EMIC/Mod DL3000 Universal Testing Machine, in which femur specimens, one at a time, were positioned horizontally on a two-support platform in the machine, with a distance between supports of 20 mm, with the femur head facing the left side and the patellar surface facing the right. The load application tip (200 kg load cell with a capacity of 2000 N)^27^ was positioned in the center of the femur and a vertical compression force was applied in the anteroposterior direction with a load application speed (force) of 0.25 mm/min until the moment of bone rupture. The values were recorded in the manufacturer's computer systems, which directly provided the maximum force values admitted by the femur. In the analysis, the bone resistance was evaluated using the force x displacement curve, analyzing the maximum force (N) admitted by the bone tissue and the stiffness (x103N/m)^28^.

### Computed microtomography analysis (micro-ct)

For micro-ct analysis, the samples were scanned and in the Data Viewer software (SkyScan, Version 1.4.4 64-bit) the images were reconstructed to fit the standard positioning for all samples and can be observed in three planes (transverse, longitudinal, and sagittal). Then, using CTAnalyser—CTAn software (2003-11SkyScan, 2012 Bruker MicroCT Version 1.12.4.0), two regions of interest (ROI) were standardized in the distal portion of the femur for trabecular bone (Upper boundary: 150 slices below the growth plate; Lower boundary: 100 slices from the upper boundary (900 µm). The volumes of interest (VOI) were automatically delimited, using the analysis software, for the trabecular bone (Boundaries of the inner border of the medullary cortex). In the trabecular portion, bone volume percentage (Tb. BV/TV), structure model index (Tb. SMI), trabecular number (Tb. N), trabecular thickness (Tb. Th), and trabecular separation (Tb. Sp) were analyzed. In the cortical portion, the volume of closed pores Po.V(cl), the volume of open pores Po.V(op), and the percentage of cortical porosity Po(tot) were evaluated^26^.

### Statistical analysis

GraphPad Prism 7.03 (GraphPad Software, La Jolla, USA) was used for statistical analysis. The homoscedasticity was assessed via the Shapiro–Wilk test to distinguish the parametric. Homocedasticity was assessed via the Shapiro–Wilk test, confirming the normal distribution of the data ANOVA one-way test was chosen to determine if there were differences among the groups. Holm Sidak post-test was performed to indicate the differences in the direct comparisons of the groups. A significance level of 5% was considered for all tests.

## Results

### Body weight and glycemic level

With the results of the average body weight, the NG and NGrvt groups had a gradual increase in weight, as expected, during the periods of the experiment. In addition, the NGrvt group maintained a lower weight gain at the time of euthanasia when compared to the NG group, while in the T2D group, there was an increase in body weight after 3 weeks of administration of the cafeteria diet. In addition, there was a greater variation in weight during the periods when compared to the T2Drvt group, which maintained a homeostasis in body weight, with no statistical difference between the periods (Table 2).

**Table 2:** Body weight average at evaluated periods.

Group	Day 0	Day 21	Day 28	Day 43	Day 73
NG	374.5 mg ± 22.53^a^	401.5 mg ± 22.86^a.b^	415.5 mg ± 21.1^b.c^	432.3 mg ± 21.56^c^	478.9 mg ± 23.73^d^
NGrvt	341.7 mg ± 24.64^a^	360.5 mg ± 21^a.b^	366.7 mg ± 22.18^a.d^	376.5 mg ± 26.86^b.c.d^	401.2 mg ± 23.68^c^
T2D	427.7 mg ± 12.33^a.c^	501 mg ± 32.15^b^	476.3 mg ± 40.16^b.c^	447.3 mg ± 42.21^a.b^	419.5 mg ± 72.61^a^
T2Drvt	435.5 mg ± 36.91^a^	493.1 mg ± 55.84^a^	483.1 mg ± 57.21^a^	481.4 mg ± 63.87^a^	517 mg ± 80.43^a^

Day 0 = Beginning of the diet; Day 21 = STZ application; Day 28 = Diabetes confirmation/start of resveratrol treatment; Day 43 = implant surgery; and Day 73 = euthanasia. The letters indicate a statistical difference between the periods.

Concerning the mean blood glucose results, the NG and NGrvt groups had hyperglycemia results, since the results were below 198 mg/dL. There was less control of blood glucose in the NGrvt group, as there was no statistical difference from the beginning to the end of the experiment. Even without a statistical difference, both diabetic groups had an increase in blood glucose 3 weeks after starting the cafeteria diet. One week after the induction of type 2 diabetes, the T2D and T2Drvt groups had a statistically significant increase in glucose levels, with values above 198 mg/dL, proving the establishment of the disease. The T2D group maintained high glycemic values with no statistical difference from the moment of induction to the moment of euthanasia. However, the T2Drvt group showed a gradual and statistically significant decrease in blood glucose after 1 week of treatment until the time of euthanasia (Table 3).

**Table 3:** Glycemia average at evaluated periods.

Group	Day 0	Day 21	Day 28	Day 43	Day 73
NG	75.7 mg/dL ± 6.584^a^	92.7 mg/dL ± 7.304^b.c^	88.5 mg/dL ± 6.346^b^	77.7 mg/dL ± 60.056^a^	97.3 mg/dL ± 6.865^c^
NGrvt	85.5 mg/dL ± 7.799^a.b^	79.75 mg/dL ± 5.119^a^	102.8 mg/dL ± 9.861^c^	95.75 mg/dL ± 11.12^c.d^	90.25 mg/dL ± 4.413^b.d^
T2D	98.3 mg/dL ± 6.255^a^	125.5 mg/dL ± 14.12^a^	384.8 mg/dL ± 83.77^b^	366.6 mg/dL ± 61.84^b^	412.2 mg/dL ± 97.54^b^
T2Drvt	95.25 mg/dL ± 5.064^a^	127.9 mg/dL ± 29.72^a^	370.9 mg/dL ± 81.11^b^	318.8 mg/dL ± 42.23^b^	227 mg/dL ± 39.93^c^

Day 0 = Beginning of the diet; Day 21 = STZ application; Day 28 = Diabetes confirmation/start of resveratrol treatment; Day 43 = implant surgery; and Day 73 = euthanasia. The letters indicate a statistical difference between the periods.

### Three-points bending testing

For the mechanical test parameters, results were obtained for maximum force; deformation at maximum force; rupture force, and deformation at rupture. For maximum force, there was a statistically significant difference between the NG vs. NGrvt group (*p* = 0.0202). The NGrvt group (137.2 N) required greater maximum force, in other words, greater force to completely break the femoral bone in the diaphysis region when compared to the NG group (115.4 N). In addition, the T2Drvt group (141.7 N) required more maximum force than the NG group, with a statistically significant difference (*p* = 0.0057). As for the maximum force deformation results, the comparison between the groups was statistically significant when comparing the T2D group (1.356 mm) vs. the T2Drvt group (1.108 mm) (*p* = 0.076). This showed that the femurs in the T2D group deformed more during the application of the maximum force compared to the T2Drvt group.

**Figure 2:**
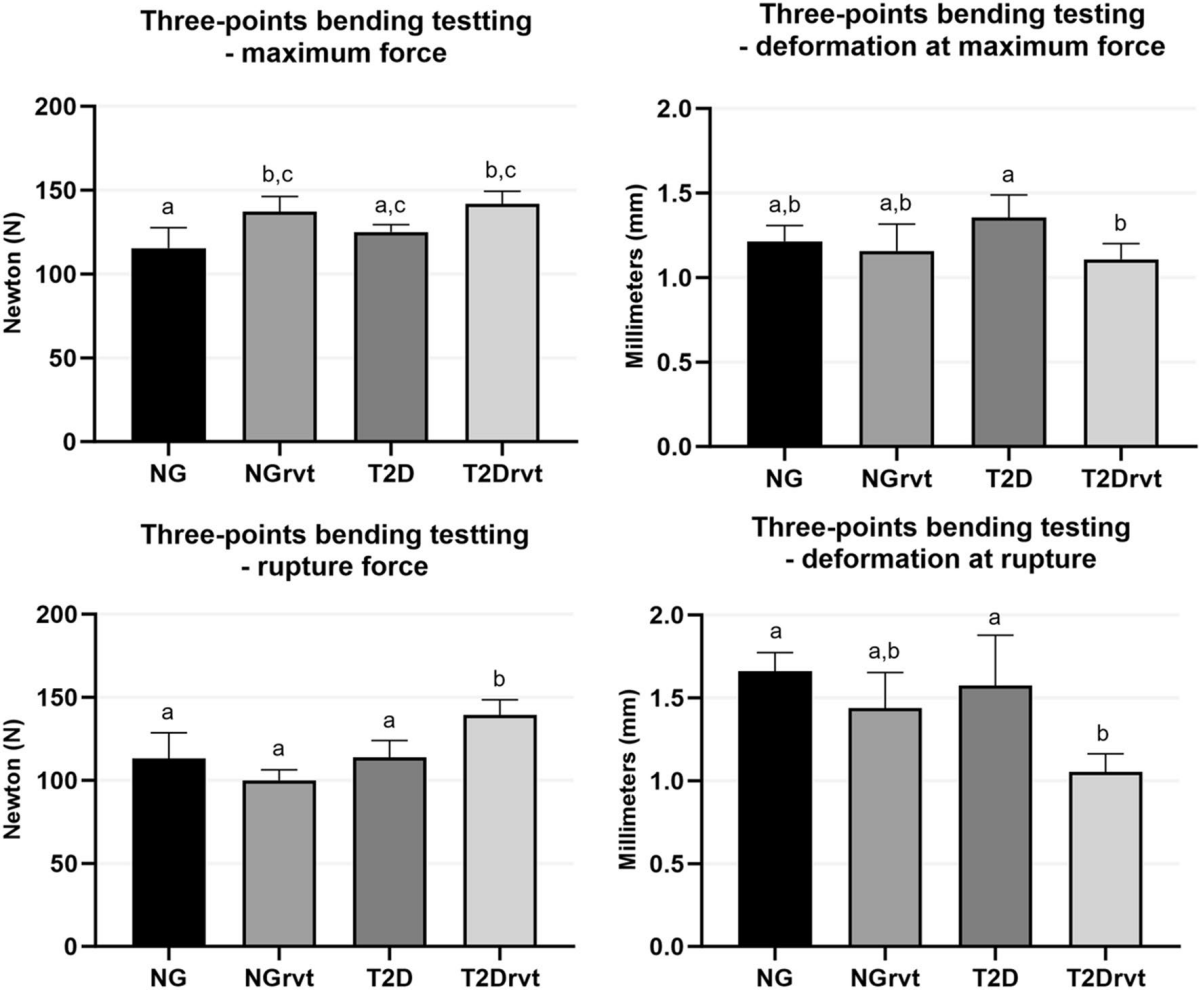
Graphs showing Three-points testing. Maximum force: The NGrvt group demanded more force to break the bone compared to the NG group, with a statistically significant difference between the group. The T2Drvt group demanded more force compared to the NG group, with a statistically significant difference between the groups. Deformation at maximum force: The T2D group deformed more compared to the T2Drvt group, with a statistically significant difference between the group. Rupture force: the T2Drvt group demanded more force to rupture the medullary when compared to all other groups, with a statistically significant difference between the group. Deformation at rupture: NG and T2D deformed more compared to T2Drvt. Symbols: Eros bar; letters indicate a statistical difference between the periods (*p* < 0.05).

In the rupture force results, there was a statistically significant difference between the NG group (113.3 N) vs. T2Drvt (139.5 N) (*p* = 0.0284), between the NGrvt group (99.96 N) vs. T2Drvt (*p* = 0.0010) and T2D group vs. T2Drvt (*p* = 0.0200). The results showed that the T2Drvt group required more force to break the femoral marrow when compared to the other groups. Finally, concerning deformation at rupture, there was a statistically significant difference between groups NG (1.66 mm) vs. T2Drvt (10.53 mm) (*p* = 0.0127) and T2D (1.575 mm) vs. T2Drvt (1.053 mm) (*p* = 0.0199). This shows that the femurs of the NG and T2D groups deformed more at the moment of rupture when compared to the T2Drvt group (Fig. 2).

### Computed microtomography analysis (micro-ct)

**Figure 3:**
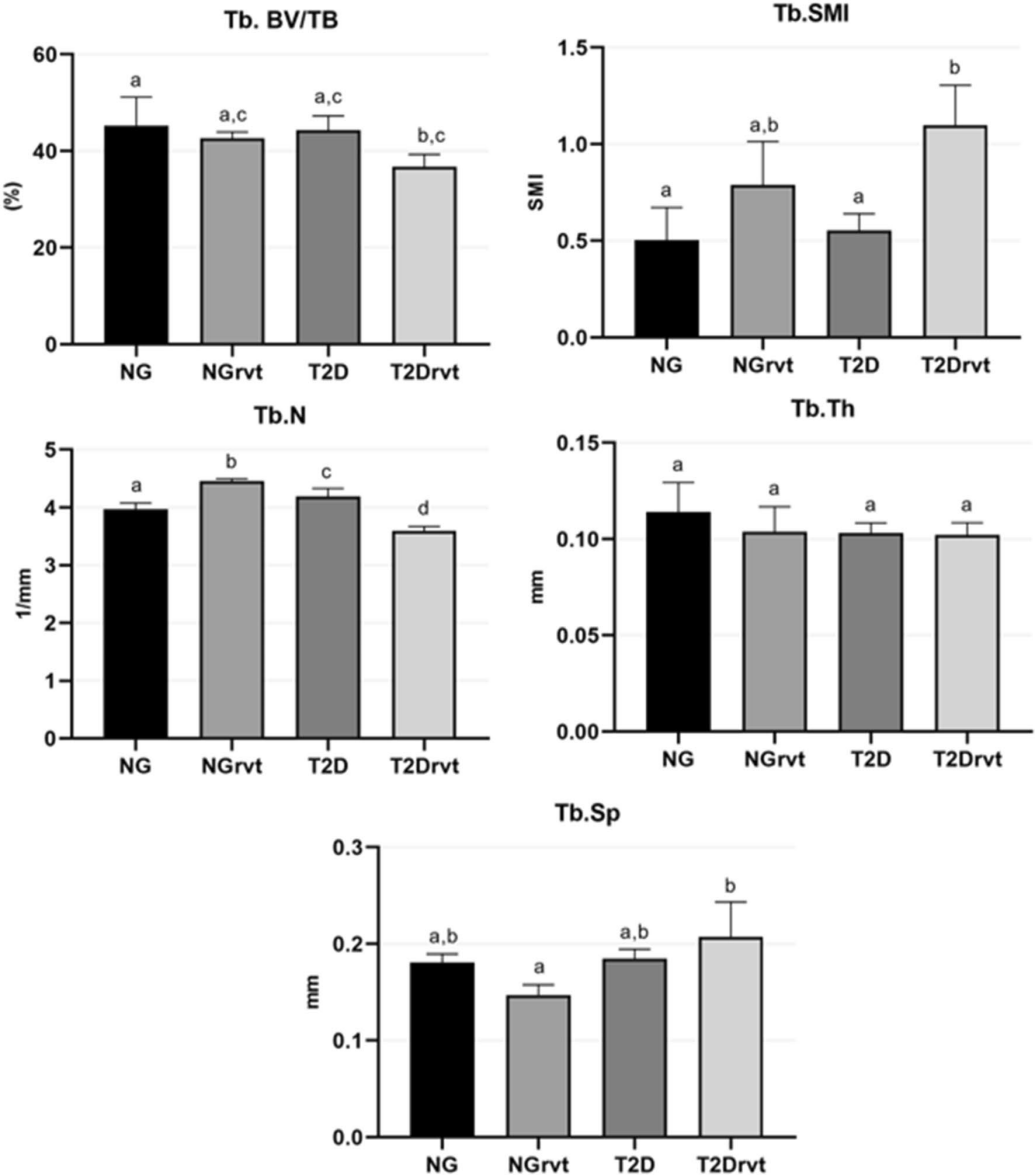
Showing Micro-CT results. BV/TV: NG group showed a higher percentage of bone volume compared to the T2Drvt group, with a statistically significant difference between the groups. Tb. SMI: T2Drvt group showed a better result compared to the NG group and T2D, with statistically significant differences between the groups. Tb. N: NGrvt group showed a higher result compared to the NG, with a statistically significant difference between the groups, and the T2Drvt group had a lower result compared to T2D, with a statistically significant difference between the groups. Tb. Th: there was no statistically significant difference between the groups. Tb. Sp: The T2Drvt group showed better results compared to the NGrvt, with statistically significant differences between the groups. Symbols: Eros bar; letters indicate a statistical difference between the periods (*p* < 0.05).

The results of the micro-ct, where the trabecular portion of the femur was assessed. For the bone volume percentage parameter (Tb. BV/TV), the NG group had a higher bone volume percentage (BV/TV = 45.24%) compared to the T2Drvt group (BV/TV = 36.72%) (*p* = 0.0468). For the structure model index parameter (Tb. SMI), the T2Drvt group (Tb. SMI = 1.098) had a higher number of rods and plates in the trabecular bone when compared to the NG group (Tb. SMI = 0.5027) (*p* = 0.0156) and also higher when compared to the T2D group (Tb. SMI = 0.5533) (*p* = 0.0249). For the number of trabeculae (Tb. N) there was a statistically significant difference among all the groups. The NG group (Tb. N = 3.969 1/mm) only had a higher number of trabeculae when compared to the T2Drvt group (3.592 1/mm) (*p* = 0.0017). In the other groups, NG had a lower number of trabeculae compared to NGrvt (Tb. N = 4.457 1/mm) (*p* = 0.0002) and T2D (Tb. N = 4.194 1/mm) (*p* = 0.485). The presence of resveratrol caused a decrease in the number of trabeculae in the T2Drvt group when compared to the NG (*p* = 0.0017), NGrvt (*p* =  < 0.0001) and T2D (*p* =  < 0.001) groups. For trabecular thickness (Tb. Th), there was no statistically significant difference for this parameter (*p* = 0.6277). For trabecular separation (Tb. Sp), the NGrvt group (Tb. Sp = 0.1471 mm) had greater separation of the trabeculae when compared to the T2Drvt group (Tb. Sp = 0.2074 mm) (*p* = 0.0077) (Fig. 3).

**Figure 4:**
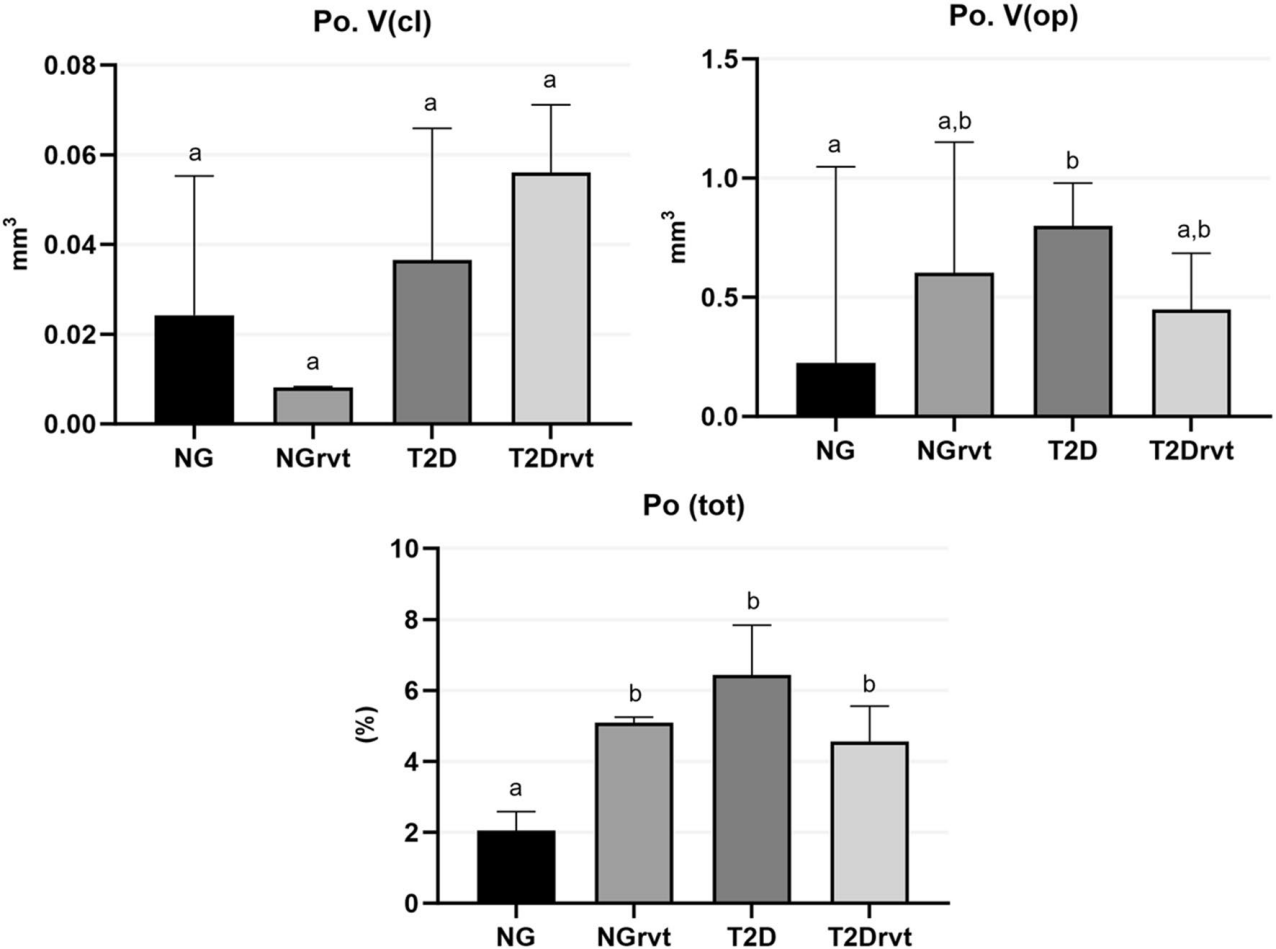
Showing Micro-CT cortical results. Po.V(cl): There was no statistical difference between the groups. Po.V(op): The T2D group showed a greater result when compared to the NG group. Po(tot): The NG group showed statistically lower results when compared to all the other groups. Symbols: Eros bar; letters indicate statistical difference between periods (*p* < 0.05).

The results of the micro-ct, in which the cortical portion of the femur was evaluated. There was no statistical difference in the volume of closed pores (Po.V(cl)) between the groups. Concerning the volume of open pores, the T2D group (Po.V(op) = 0.799 mm^3^) had a greater volume of open pores in the cortical bone when compared to the NG group (Po.V(op) = 0.2246 mm^3^) (*p* = 0.0087). For the percentage of cortical porosity, the results were lower for the NG group when compared to all the other groups. The NG group obtained lower values (Po (tot) = 2.025%) for the percentage of cortical porosity when compared to the NGrvt (Po (tot) = 5.086%) (*p* = 0.0143), T2D (Po (tot) = 6.431%) (*p* = 0.0016) and T2Drvt (Po (tot) = 4.558%) (*p* = 0.376) groups (Fig. 4).

### Biomechanical analysis of counter-toque

**Figure 5:**
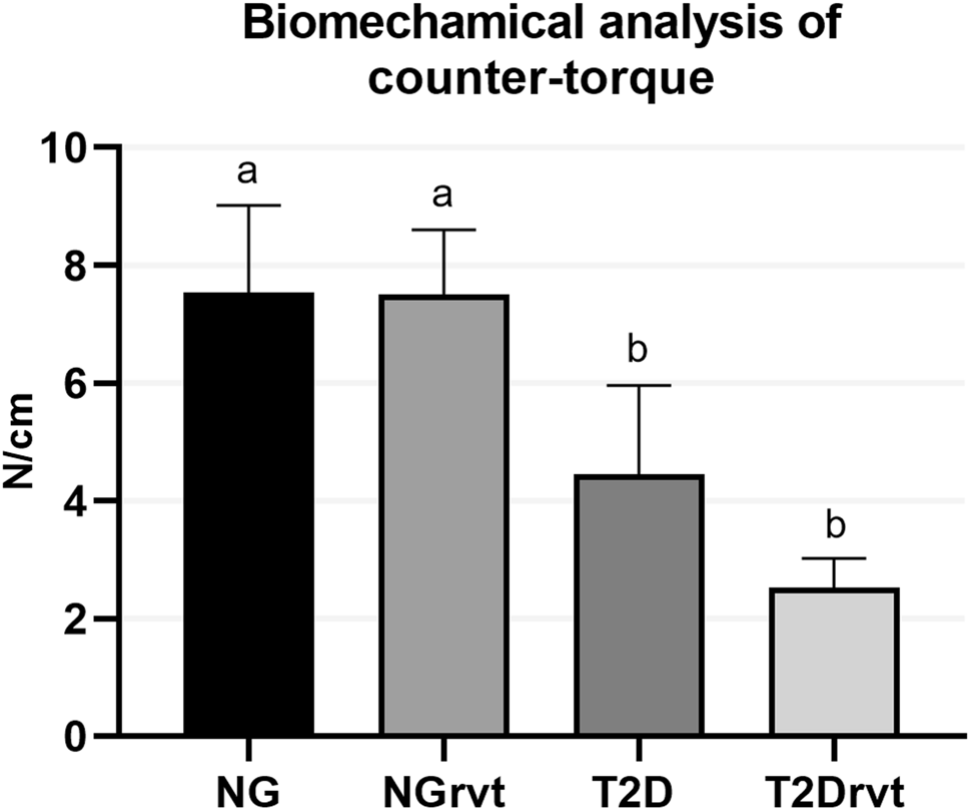
Graphs showing Biomechanical analysis of counter-torque. The normoglycemic groups (NG and NGrvt) showed a higher counter-torque value when compared to the groups with type 2 diabetes (T2D and T2Drvt). Symbols: Eros bar; letters indicate a statistical difference between the periods (*p* < 0.05).

For the biomechanical parameters of counter-torque analysis, there was only a statistical difference between the normoglycemic and type 2 diabetic groups, regardless of the administration of resveratrol. The NG group (7.54 N/cm) showed a higher counter-torque value than the T2D (4.45 N/cm) (*p* = 0.0151) and T2Drvt (2.252 N/cm) groups (*p* = 0.0003). The NGrvt group (7.5 N/cm) showed a higher counter-torque value when compared to the T2D (*p* = 0.0164) and T2Drvt (0.0004) groups. As for the diabetic groups, there was also no statistical difference between them (*p* = 0.1582) (Fig. 5).

## Discussion

In addition to being involved in the etiopathogenesis of type 2 diabetes, obesity also has a pathophysiological impact^29^, being one of the main risk factors, along with the presence of foods rich in sugar, fat, and sodium^30^, the latter mimicked in this study by the cafeteria diet. Resveratrol, the phytochemical used in this study, has been widely studied^31^, as it is a great candidate for adjuvant therapy of type 2 diabetes^32–35^. Studies have shown that resveratrol has a positive effect on lipid homeostasis and the reduction of fat mass^36^, which can be partially explained by both the activation of lipolysis through adipose triglyceride lipase and the inhibition of lipogenesis^37–40^. This action on lipid hemostasis was observed in this study, as the NGrvt group maintained a lower weight gain when comparing the first to the last day of the experiment compared to the NG group. On the other hand, in the diabetic groups that received the cafeteria diet, T2D showed the greatest statistical variation in body weight during the experimental period. There was a significant increase in weight after starting the cafeteria diet and a slight reduction in weight after the injection of streptozotocin, which may be related to the application of the drug, as well as to acute hyperglycemia^22^. Unlike the T2Drvt group, which received treatment with resveratrol, which showed weight homeostasis, represented by no statistical difference between the dates. This shows the effect of resveratrol on weight stability, energy expenditure, and reduction of adiposity^41^.

Concerning glycemic levels, both normoglycemic groups showed the expected normal values, below 100 mg/dl. On the other hand, in the diabetic groups, only the cafeteria diet, although with no statistical difference, was able to raise glucose levels. This shows that a nutrient-poor diet is a risk factor because it increases glycemic patterns^10,30^. After the induction of type 2 diabetes, there was a significant increase in blood glucose in the T2D and T2Drvt animals, with values higher than 198 mg/dL, proving the onset of the disease and demonstrating the efficacy of streptozotocin in this animal model of type 2 diabetes. In this study, in addition to observing the beneficial action of the systemic administration of resveratrol on weight, there was an action mainly on glycemic patterns. The resveratrol, from the start of its administration in the T2Drvt group, was responsible for drastically and statistically reducing glycemic levels until the end of the experiment. Unlike the T2D group, which maintained high blood glucose levels after induction until euthanasia. These results corroborate studies that show improved insulin sensitivity in type 2 diabetic rats when resveratrol is administered^42–44^, through the potent activation of Sirt1, increasing insulin sensitivity and protecting against metabolic damage. Studies in the literature show that activation of AMP-activated protein kinase (AMPK) is used to mediate some of the effects of resveratrol in regulating insulin sensitivity and insulin secretion in pancreatic β-cells and in increasing glucose uptake^45^.

The flexion analysis showed the beneficial effects of resveratrol in the normoglycemic group and also in the diabetic animals. In the NGrvt group, resveratrol contributed to increased stiffness, which required greater force to completely break the bone when compared to the untreated normoglycemic group. In the T2Drvt group, greater force was required to break the entire medullary femurs when compared to all the other groups. This may be explained by the additional beneficial effects of resveratrol and the greater bone density in type 2 diabetics^46^. These results corroborate studies showing that resveratrol, even under hyperglycemic conditions, is capable of modulating osteogenic and osteoclastogenic mechanisms in the bone remodeling process during healing, as well as preventing the loss of bone density^47^. It can also be seen that resveratrol reduces bone deformation in the T2Drvt group, both in terms of deformation at maximum force and deformation at break. This shows that the bone in the T2Drvt group has a stiffness improvement, requiring an increasing strength to break the femur. Results express the promising effects of resveratrol in improving the mechanical properties of bone^48^ and its protective effects that seem to be mediated by the increased bone formation of osteoblasts, possibly due to reduced inflammation^49^.

In the micro-ct analysis, in the medullary portion, the T2Drvt group showed a lower number of trabeculae, with greater separation between them. However, this data did not influence trabecular thickness, possibly because it had a higher structure model index, which means a higher relative prevalence of plates and rods. As there is a greater separation of these trabeculae, it is to be expected that the bone volume would be smaller in T2Drvt, as it is a more porous bone. As for the cortical portion, type 2 diabetes negatively affected the cortical structure of the bone, with a greater volume of open pores and a higher percentage of cortical porosity when compared to the normoglycemic group. These results are already known in the literature, where the most consistent alteration of diabetes is the increase in cortical bone porosity^50^. This damage is attributed to the morphology and function of osteocytes, which are known to act as organizers of bone metabolism^51^. About the DM2 groups, resveratrol was not statistically capable of improving cortical quality. However, these structural changes did not have a sufficient influence on the biomechanics of the femur. In the three-point bending test, there was no loss of strength in the presence of type 2 diabetes.

The biomechanical analysis using the three-point bending test and micro-ct showed that the presence of type 2 diabetes could not cause significant changes in the biomechanics and microstructure of the long bones. The increased risk of fracture in long bones is more evident in the case of type 1 diabetes, as they have reduced bone mass^52^. This factor is not found in type 2 diabetes, where patients have greater bone density. This is due to the anabolic effect of hyperinsulinemia, which favors osteoblastic activity, leading to increased bone density^46^. In addition, studies report that a higher incidence of fracture in type 2 diabetic patients is related to chronic complications, which are responsible for causing greater functional limitation^52^, as well as the use of some medications that can reduce bone density^53^.

The results show the superiority of the normoglycemic groups in the osseointegration of the bone/implant interface in the tibia compared to the diabetic groups, demonstrating the relevance of the systemic interference of type 2 diabetes in bone metabolism when there is tissue injury. In normal repair, tissues progress through discrete phases, including hemostasis, inflammation, proliferation, and remodeling, but in diabetes, progression through these phases is impaired, resulting in a sustained inflammatory state and dysfunctional epithelialization in the wound^54^. There are also alterations in microvascularization which decrease the immune response, reduce bone remodeling, and compromise the vascularization of the flap, delaying healing and providing a gateway for infections^55^. Although some studies have highlighted the benefits of using resveratrol as a therapeutic agent to improve peri-implant bone healing in the presence of diabetes mellitus^56^, the administration of resveratrol made no statistical difference in this study.

The results in long bones revealed promising effects of resveratrol in improving bone microstructure and mechanical properties, being able to improve strength and resistance parameters in femurs. Scientific evidence shows that type 2 diabetes is detrimental to the bone metabolism of long bones, however in this study, little evidence was obtained. It was possible to observe that type 2 diabetes is more harmful when there is tissue damage, such as in the presence of implants. Peri-implant osseointegration in type 2 diabetic animals was worse when compared to control groups. This is a problem that arises in dental practices when surgical procedures such as implant installation are required, as osseointegration may be impaired. The systemic administration of resveratrol was unable to reverse the peri-implant results. However, it is very effective in etiopathogenesis, acting on weight homeostasis and especially in reducing glycemic levels in diabetic rats. Therefore, new studies will be carried out to evaluate resveratrol locally, by functionalizing implants with resveratrol.

## Conclusion

Resveratrol acted positively in type 2 diabetes, reducing hyperglycemia and acting on weight homeostasis. Resveratrol in the long bones of type 2 diabetics showed positive biomechanical effects on strength and endurance parameters. However, resveratrol was unable to improve the microstructure of diabetic long bones and its systemic administration did not bring positive results in the quality of osseointegration at the bone-implant interface, when tissue damage and the inflammatory process were already present. Revealing that resveratrol supplementation can be used in conjunction with dietary therapy and in a preventive manner. And exposing the need for further work to detect its local effects on the implant surface.

## Data Availability

The datasets generated and/or analyzed are available from the corresponding author upon reasonable request.
